# P019 Below the lower limit - trending lifelong alkaline phosphatase leading to a diagnosis of adult hypophosphatasia

**DOI:** 10.1093/rap/rkag113.049

**Published:** 2026-11-03

**Authors:** Julia McEvoy, Katie Moss, Catherine McGrath

**Affiliations:** Frimley Park Health NHS Foundation Trust, Frimley, United Kingdom; St George’s University Hospitals NHS Foundation Trust, London, United Kingdom; Frimley Park Health NHS Foundation Trust, Frimley, United Kingdom

## Abstract

**Introduction:**

Hypophosphatasia (HPP) is a rare inherited metabolic bone disorder caused by variants in the *ALPL* gene. The *ALPL* gene encodes the tissue-nonspecific alkaline phosphatase (TNSALP) enzyme. TNSALP deficiency leads to extracellular accumulation of inorganic pyrophosphate, a potent inhibitor of bone mineralization. Clinical manifestations of HPP are protean ranging from severe perinatal disease to milder adult forms. In HPP, anti-resorptive medications such as bisphosphonates and denosumab are avoided along with excess calcium supplements.

Recognition of the significance of persistently low serum alkaline phosphatase (ALP) is vital. We present a case of adult HPP incidentally identified in a new patient.

**Case description:**

In a new patient encounter for Sjogren’s disease, note was made of lifelong low alkaline phosphatase in a 50 y.o. woman (usually in the range 15-25 U/L, normal >30 U/L). History was significant for arthroscopic debridement of both hips for bone spurs (3 in total). She had a diagnosis of congenital anteversion of the left femoral neck and underwent a subtrochanteric femoral derotational osteotomy in her 30’s. She had a long period on crutches with delayed bone healing around the surgical pin which was subsequently removed after a year. She reported dental problems and multiple fillings before the age of 15. There was a history of chronic musculoskeletal pain (particularly lateral hips), muscle weakness and profound fatigue. She reported multiple toe fractures relating to dance. Bone density on DEXA scan was supranormal.

She did not have children and family history included multiple miscarriages in her mother. A sister had Crohn’s disease.

Common causes of low alkaline phosphatase were investigated including nutritional deficiencies for zinc and magnesium, vitamin B12 and vitamin B6. Malabsorption syndromes including coeliac disease and inflammatory bowel disease were excluded. She has difficult to control hypothyroidism and thyroid indices were at times out of range (both hyper- and hypo-thyroid). Hypothyroidism can lead to slower metabolism and low bone turnover leading to decreased ALP production. She was not on oestrogen containing medications nor anti-resorptive bone medications. Fasting vitamin B6 was elevated. Urinary phosphoethanolamine was not available locally (expected to be elevated).

Referral was made to a tertiary metabolic bone service in London for further assessment and management. Genetic analysis demonstrated an *ALPL* heterozygous genetic mutation supporting the diagnosis of adult HPP.

The patient subsequently underwent specialist review with recommendations for ongoing monitoring, optimisation of bone health, avoidance of inappropriate anti-resorptive therapy and family counselling/testing.

**Discussion:**

This case highlights the importance of recognising persistently low ALP. In routine practice, low ALP may be overlooked because isolated results below the lower limits are often considered clinically insignificant. However, repeated low measurements should prompt evaluation for secondary causes and consideration of inherited conditions such as HPP.

HPP is an inborn error of metabolism that directly or indirectly affects multiple organ systems. Adult HPP presentations are particularly challenging because symptoms are often non-specific and may include musculoskeletal pain, stress fractures, osteomalacia, dental complications, fatigue or may be entirely incidental. Results need careful interpretation and, for example, bone density from DEXA scans can be misleading with build-up of unmineralized bone osteoid giving a normal result despite a higher incidence of fractures/pseudofractures.

Diagnosis relies on integrating clinical presentation, persistently low ALP, supportive biochemical markers such as elevated vitamin B6 and urinary phosphoethanolamine and confirmation through ALPL genetic testing. Genotype-phenotype correlations are highly variable.

Our patient has concurrent medical conditions associated with musculoskeletal symptoms and fatigue. It would be easy to apportion her symptoms to the existing diagnoses and stop there. With her diagnosis of HPP, she has a greater understanding of the source of her symptoms and feels that things make more sense overall. She did not require enzyme replacement therapy in the form of asfotase alfa but it was discussed.

Recognition of adult HPP has important therapeutic implications. Patients may otherwise receive alternative diagnoses such as osteoporosis and be exposed to antiresorptive therapies that may worsen skeletal complications in HPP. Early identification allows specialist input, surveillance for complications, consideration of family screening and informed management decisions.

This case demonstrates that a simple laboratory abnormality can provide the first clue to a rare inherited metabolic disorder and reinforces the value of reviewing biochemical trends rather than isolated test results.

**Key learning points:**

1. Persistently low ALP warrants further investigation. Low serum alkaline phosphatase should not automatically be dismissed as clinically insignificant, particularly when repeatedly demonstrated over time

2. Exclude secondary causes before pursuing rare diagnoses. Causes including malnutrition, hypothyroidism, coeliac disease, vitamin and mineral deficiencies and medication effects should be considered and excluded systematically

3. Specialist referral is valuable. Referral to a tertiary metabolic bone centre facilitates confirmation of diagnosis, interpretation of genetic findings, multidisciplinary management and counselling and potentially access to specialist medications (including enzyme replacement therapy).

4. Early recognition can avoid complications further down the line. Correct identification of hypophosphatasia may avoid inappropriate therapies and enable tailored approaches to bone health and long-term monitoring

